# Storage time of intraoperative transfused allogeneic red blood cells is not associated with new-onset postoperative atrial fibrillation in cardiac surgery

**DOI:** 10.1371/journal.pone.0172726

**Published:** 2017-02-22

**Authors:** Jiwei Gu, Regitze Kuhr Skals, Christian Torp-Pedersen, Søren Lundbye-Christensen, Carl-Johan Jakobsen, John Bæch, Mikkel Steen Petersen, Jan Jesper Andreasen

**Affiliations:** 1 Department of Cardiovascular Surgery, Heart Centre of General Hospital, Ningxia Medical University, Yinchuan, Ningxia, PR China; 2 Department of Clinical Medicine, Aalborg University, Aalborg, Denmark; 3 Atrial Fibrillation Study Group, Aalborg University Hospital, Aalborg, Denmark; 4 Unit of Epidemiology and Biostatistics, Aalborg University Hospital, Aalborg, Denmark; 5 Unit of Clinical Biostatistics, Aalborg University Hospital, Aalborg, Denmark; 6 Department of Anaesthesiology, Aarhus University Hospital, Aarhus, Denmark; 7 Department of Clinical Immunology, Aalborg University Hospital, Aalborg, Denmark; 8 Department of Clinical Immunology, Aarhus University Hospital, Aarhus, Denmark; 9 Department of Cardiothoracic Surgery, Aalborg University Hospital, Aalborg, Denmark; Goethe University Medical School, GERMANY

## Abstract

**Background:**

Allogeneic red blood cell (RBC) transfusion has been associated with new-onset postoperative atrial fibrillation (POAF) following cardiac surgery. Prolonged storage time of RBC may increase the risk. The primary aim of the study was to evaluate whether the storage time of RBC is associated with development of POAF.

**Materials and methods:**

Pre-, per- and postoperative data were retrieved from the Western Denmark Heart Registry and local blood banks regarding patients who underwent coronary artery bypass surgery, valve surgery or combined procedures in Aalborg or Aarhus University Hospital during 2010–2014. Multiple logistic regression was used to determine the risk of POAF according to transfusion of RBC on the day of surgery. Furthermore, we determined trend in storage time of RBC according to risk of POAF using restricted cubic splines. Patients with a history of preoperative atrial fibrillation, patients who received transfusions preoperative and patients who died at the day of surgery were among excluded patients.

**Results:**

A total of 2,978 patients with a mean age of 66.4 years were included and 609 patients (21%) received RBC transfusion on the day of surgery. POAF developed in 752 patients (25%) and transfused patients were at an increased risk compared with non-transfused patients (adjusted Odds Ratios for patients receiving RBC: 1.37; 95% CI: 1.11–1.69, *P*-value = 0.004). However, RBC transfusion was not necessarily the cause of POAF and may only be a marker for development of POAF. There was no significant association between storage time of RBC and POAF.

**Conclusions:**

In contrast to intraoperative allogeneic RBC transfusion in general, increased storage time of RBC is not associated with development of POAF in cardiac surgery.

## Introduction

Postoperative new-onset atrial fibrillation (POAF) is the most common arrhythmia after cardiac surgery with a reported incidence of approximately 20–60%, depending on definition, type of surgeries and the methods used for identifying the diagnosis [1–3].

Despite decades of surgical, anaesthetic, and medical advances, rates of POAF following cardiac surgery remain largely unchanged. Because POAF is associated with increased morbidity, mortality and increased resource utility [1,2,4,5], there is a continuous awareness regarding possibilities to predict, prevent and treat POAF [6,7]. Several patient-related and procedure-related risk factors and predictors of POAF have been described [8–10], but additional information on potential new predictors of POAF is needed in order to identify more patients at risk.

The pathogenesis of atrial fibrillation including POAF seems to be multifactorial [10–12] and also allogeneic red blood cell (RBC) transfusion has been associated with development of POAF [13,14]. RBC transfusion is associated with development of a postoperative systemic inflammatory response syndrome [15], and inflammation as a causative pathophysiologic element of POAF is supported by the literature [16,17].

Several observational and a few randomized studies have evaluated whether storage time in the blood bank prior to transfusion is associated with an increased the risk of adverse outcomes following cardiac surgery, but results are controversial [18,19]. Some observational studies reported that storage of RBC for >2 weeks were associated with e.g. infections, renal and respiratory disorders [19], however, a randomized study failed to support these findings focusing on multiple organ dysfunction [18]. A plausible explanation for adverse outcomes associated with older blood are the well described structural and functional changes that occur in stored RBCs over time [20].

To our knowledge only one study have reported on the development of POAF following cardiac surgery in relation to transfusion of “older” versus “younger” blood [21]. The primary aim of the present study was therefore to evaluate whether storage time of transfused RBC is associated with development of POAF in patients undergoing cardiac surgery. Furthermore, we aimed at investigating if RBC in general is associated with an increased risk of POAF. We hypothesized that both RBC transfusion in general and increased storage time of transfused RBC are associated with an increased risk of POAF.

## Materials and methods

### Study design

This study is an analysis of consecutive patients undergoing coronary artery bypass grafting (CABG), valve surgery or combinations between 2010–2014 in Aalborg or Aarhus University Hospitals located in the Central and North Denmark Regions. These hospitals serve a population of approximately 2.5 million persons equal to 33% of the total population in Denmark.

The Danish National Health Service provides tax-funded medical care for all Danish residents, and due to the unique Central Personal Registry number assigned to each Danish citizen at birth and to residents on immigration, we were able to perform a linkage between hospital administrative systems and databases at an individual level. The study was approved by the Danish Data Protection Agency (record numbers: 2008-58-0028 and 2014-41-3419). The Danish Data Protection Agency does not require researchers to obtain written consent from participants for their medical records to be used in observational epidemiological studies.

### Patients

From the hospital administrative systems and the Western Denmark Heart Registry (WDHR) [22] we identified all patients (>15 years) who underwent either on- or off-pump CABG, conventional valve or combined surgeries in the two hospitals between January 1, 2010 to December 31, 2014. The WDHR holds prospectively collected information regarding patients, procedures and in-hospital postoperative outcomes for all patients undergoing invasive cardiac procedures in Western Denmark. We excluded patients with a history of atrial fibrillation or flutter prior to surgery and patients with an invalid personal registration number. Patients who received RBC transfusions prior to surgery or postoperative at another day but the day of surgery were excluded in order to ensure that transfusion of RBC was given prior to the development of the outcome of interest i.e. POAF. Patients who died on the day of surgery were excluded, due to the inability to tell whether they would develop the outcome of interest or not.

### Data on blood transfusions

Information in relation to RBC transfusions delivered to the patients were retrieved from local databases in the blood banks (number of units, date of delivery, storage time in days prior to transfusion). Patients were classified as having received either none or the actual number of RBC units delivered.

### Perioperative management and surgical techniques

Beta-blockers and statins were in general not withdrawn before surgery. For prevention of POAF, amiodarone was administered to approximately 20–25% of the patients in Aarhus University Hospital intravenously over 20 minutes on the first postoperative day together with an oral dose of 600 mg of amiodarone twice daily during the initial five postoperative days. Indications for this preventive treatment were related to some surgeons. Anaesthesiology and surgical techniques were those routinely used in the participating hospitals. In brief, a decision to perform off-pump CABG was dependent on the surgeon. The core temperature was allowed to drift a few degrees when cardiopulmonary bypass was used. The left internal mammary artery was used for grafting whenever possible if the left anterior descending artery was to be grafted. Blood conservation techniques varied slightly between the participating hospitals. A decision to stop anti-platelet drugs prior to surgery was based upon recommendations [23] published before the time period during which patients underwent surgery taking the coronary pathology and urgency of surgery into consideration. Vitamin K antagonists were usually withheld 2–3 days preoperative. The majority of patients received peroperative anti-fibrinolytic treatment with tranexamic acid. Residual blood from the cardiopulmonary bypass circuit was routinely re-transfused to the patients at the end of surgery. Blood products were given at the discretion of the attending anaesthesiologist or surgeon. Local transfusion guidelines existed in both hospitals and all RBC transfusions delivered were leucocyte-depleted. Auto-transfusion was not used in Aalborg Hospital. Aspirin therapy was in general initiated or resumed in the morning the day after surgery. At the same time, thrombosis prophylaxis with low molecular weight heparin was initiated and administered for at least five days. Patients were routinely discharged on day 6–8 postoperative if no postoperative complications developed.

### Data on POAF

On the day of discharge or a few days later a diagnosis of POAF was entered directly into the WDHR or registered on a database sheet for later registration in the database. Whether POAF developed or not was based on written information in the patient record and available ECGs. In general, new-onset POAF was defined as atrial fibrillation or atrial flutter occurring postoperative during hospitalization regardless of duration and whether the patient required treatment due to POAF. All patients were monitored routinely postoperative with continuously ECG for two-three days in Aalborg University Hospital and for one day in Aarhus University Hospital. Thereafter conventional ECGs were obtained if any clinical suspicion based on pulse palpation and symptoms of POAF developed. All patients had an ECG taken prior to discharge. POAF, which was the main outcome of interest, was considered a binary outcome during hospitalization from one day postoperatively as we had no information regarding the actual day POAF developed.

### Data on covariates

Pre- per- and postoperative data including potential confound data that may be associated with both requirements for transfusions and POAF were retrieved from the WDHR. Preoperative data were patients age, gender, body mass index (BMI), diabetes mellitus, hypertension (= medical treatment), chronic obstructive pulmonary disease (COPD) (= medical treatment), a history of previous myocardial infarction within three months preoperative, left ventricular ejection fraction (LVEF), p-creatinine, need of dialysis, preoperative intake of anti-platelet drugs (acetylsalicylic acid and clopidogrel) within five days preoperative and β-blockers and angiotensin-converting enzyme (ACE) inhibitors as well as the logistic EuroSCORE II. Peroperative data were: Type of surgery, use of cardio-pulmonary bypass and place of surgery i.e. Aarhus or Aalborg University Hospital.

### Statistical analyses

Transfused and non-transfused patients were compared using descriptive statistics including mean, standard deviations, and percentage of patients when appropriate. Differences between the study groups were analysed using chi-squared tests for categorical variables, and analysis of variance for continuous variables. A *P*-value <0.05 was considered statistically significant.

Multiple logistic regression was used to determine the risk of developing POAF according to transfusion of RBC on the day of surgery, as well as storage time of RBC and dose-dependence for the subpopulation of patients who received RBC. Odds ratios (OR) and probability i.e. risks of developing POAF were adjusted for patient age, gender, presence of COPD, presence of peripheral arteriosclerosis, preoperative treatment with β-blocker, ACE- inhibitor and calcium antagonists and type of surgery including on- and off-pump regarding the CABG patients. For the subpopulation who received RBC transfusions, the estimates were also adjusted for the number of RBC transfusions received. Confounding factors adjusted for were pre-specified based on the literature.

Restricted cubic splines were used to determine whether another categorization than 14 days would be better to describe a possible association between storage time of RBC and POAF for the subpopulation of patients who received RBC. Two analyses were performed: First, if a patient did receive more than one unit of RBC, the oldest one was used for analysis; secondly, the storage time averaged across all RBC units received in each patient was used. The quantiles used for creating the knots were 10%, 50% and 90% of the observed storage times in both analyses. The knots were moved one day to the left and three days to the right as a sensitivity analysis of placement of the knots. Due to the limitation on information regarding date of development of POAF, statistical models were made based on the assumption that patients lived long enough to develop POAF, assuming POAF most often develop within day 2–5 postoperative. Statistical analyses were performed using the software R (R Core Team version 3.2.2).

## Results

A total of 4,766 patients underwent surgery during the study period. Among these, 2,978 patients with a mean age of 66.4 years (range 15–91 year) were included. Table 1 shows the baseline characteristics among patients included according to development of POAF.

**Table 1:** Baseline patient and operative characteristics.

Variable		No-POAF(n = 2,226)	POAF (n = 752)	Total (n = 2,978)	*P*-value
Male (%)		1,737 (78.0)	552 (73.4)	2,289 (76.9)	>0.0001
Age					
	Mean (SD), years	65.2 (10.8)	70.0 (9.4)	66.4 (10.7)	<0.0001
	Range, years	15–91	24–91	15–91	
Age grouped by years (%)					
	<60	618 (27.8)	97 (12.9)	715 (24.0)	
	61–66	532 (23.9)	141 (18.8)	673 (22.6)	
	67–73	541 (24.3)	212 (28.2)	753 (25.3)	
	>73	535 (24.0)	302 (40.2)	837 (28.1)	<0.0001
BMI					
	Mean (SD)	27.2 (4.3)	26.9 (4.2)	27.1 (4.3)	0.151
	>40	19 (0.9)	1 (0.1)	20 (0.7)	0.067
AMI (%)					
	Within three months preoperative	415 (18.7)	110 (14.7)	525 (17.7)	0.014
	(Missing)	11	2	13	
LVEF (%)					0.584
	>50	1542 (72.5)	511 (71.3)	2053 (72.2)	
	30–50	470 (22.1)	171 (23.8)	641 (22.5)	
	<30	114 (5.4)	35 (4.9)	149 (5.2)	
	(Missing)	100	35	135	
Diabetes mellitus (%)					
	Diabetes mellitus	74 (3.4)	13 (1.8)	87 (3.0)	0.033
	(Missing)	62	19	81	
COPD (%)		161 (7.2)	101 (13.4)	262 (8.8)	<0.0001
EuroSCORE (Logistic II)					
	Mean (SD)	2.6 (5.6)	3.0 (5.4)	2.7 (5.6)	0.202
	(Missing)	1117	412	1529	
Peripheral arteriosclerosis (%)		161 (7.2)	59 (7.8)	192 (6.4)	0.085
Stroke (%)					
	Stroke	91 (4.1)	45 (6.0)	136 (4.6)	0.039
	(Missing)	5	3	8	
s-Creatinin (%)					
	>200 μmol/L	33 (1.5)	10 (1.3)	43 (1.5)	0.881
	(Missing)	29	4	33	
Dialysis (%)					
	Dialysis	16 (0.7)	8 (1.1)	24 (0.8)	0.494
	(Missing)	4	3	7	
NYHA class (%)					
	>2	228 (20.6)	82 (24.1)	310 (21.4)	0.186
	(Missing)	1117	412	1529	
Medications (%)					
	β-Blocker	1,208 (54.3)	417 (55.5)	1,625 (54.6)	0.602
	Calcium antagonist	536 (24.1)	200 (26.6)	736 (24.7)	0.182
	ACE inhibitor	883 (39.7)	302 (40.2)	1,185 (39.8)	0.845
Valve surgery (%)		607 (27.3)	288 (38.3)	895 (30.1)	<0.0001
CABG (%)					
	Off-pump	448 (20.1)	126 (16.1)	574 (19.3)	
	On-pump	990 (44.5)	258 (34.3)	1,248 (41.9)	<0.0001
Combined surgery (%)		181 (8.1)	80 (10.6)	261 (8.8)	0.043

POAF: New onset postoperative atrial fibrillation; BMI: Body Mass Index; SD: Standard deviation; AMI: Acute myocardial infarction; LVEF: Left ventricular ejection fraction; COPD: Chronic obstructive pulmonary disease; NYHA: New York Heart Association; ACE: angiotensin-converting enzyme; CABG: Coronary artery bypass grafting.

**Fig 1:**
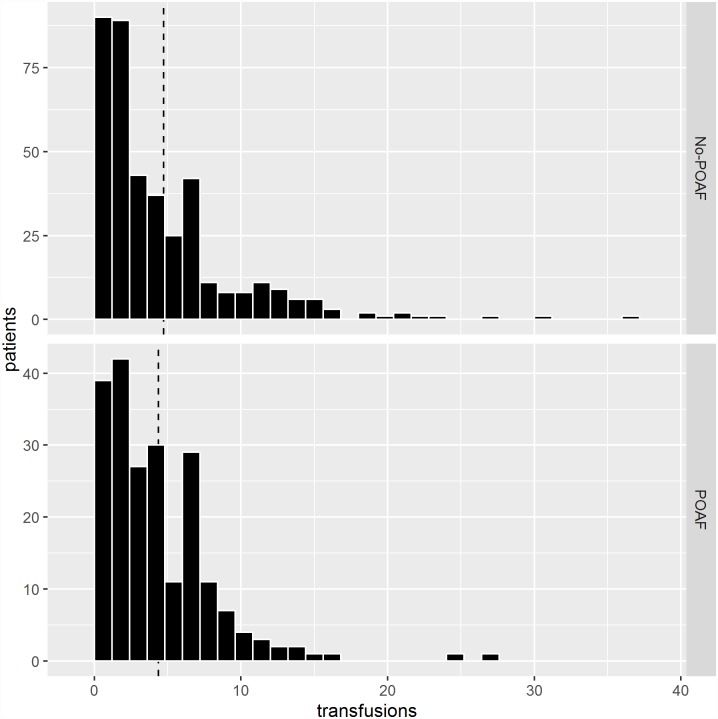
Number of red blood cell units transfused on the day of surgery to each patient according to development of postoperative atrial fibrillation. Dashed line represents the average number of transfused units for each patient.

POAF developed in 752 patients (25%). Patients who developed POAF were e.g. more likely to be older, female, and to suffer from comorbidities like COPD and previous stroke. Furthermore, valve or combined surgery were more common among patients who developed POAF. In all, 609 patients (20.4%) received ≥1 transfusions of RBC on the day of surgery. The numbers of transfused units to each patient are shown in Fig 1.

Transfused patients were more likely to be older compared to non-transfused patients (*P-*value <0.0001).

**Fig 2:**
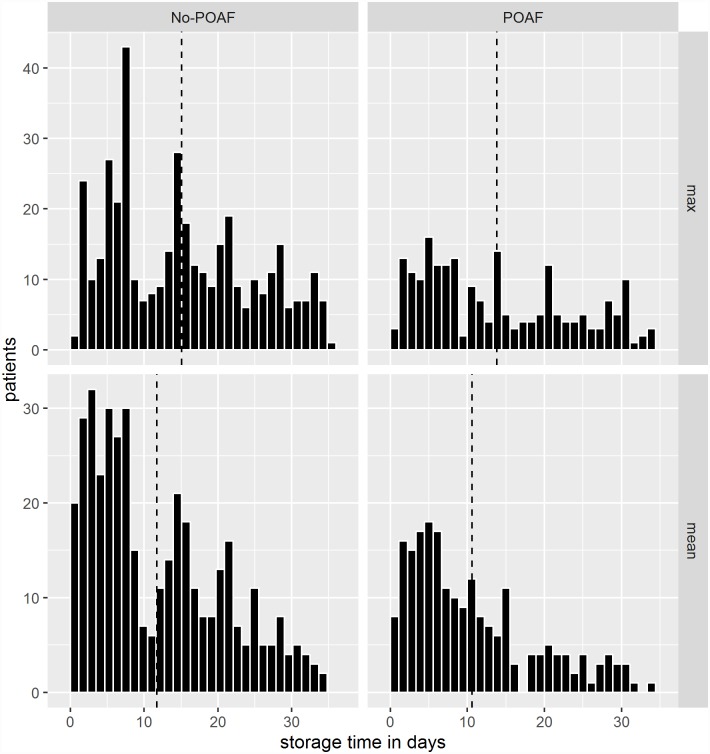
Distribution of maximum and mean storage time of red blood cell units transfused according to development of postoperative atrial fibrillation. Dashed lines represent the average storage time.

Fig 2 shows the distribution of the maximal and mean storage time of RBC units transfused to patients. The dashed line represents the average storage time.

Transfused patients were at an increased risk for development of POAF compared with non-transfused patients. The unadjusted and adjusted OR for development of POAF in transfused patients compared with non-transfused patients were 1.79; 95% CI: 1.48–2.17 (*P*-value ≤0.001) and 1.37; 95% CI: 1.11–1.69 (*P*-value = 0.004), respectively. An increased risk by dose was found for up to 4–6 units of RBC cells with an OR of 1.62; 95% CI: 1.08–2.42 for patients receiving 4–6 units of RBC compared with patients receiving 1–3 units of RBC. No interaction between hospital and transfusion was found in the analyses.

Unadjusted and adjusted OR for development of POAF in transfused patients dividing transfused patients into two groups whether they were transfused with RBC stored for either <14 days or ≥14 days compared with non-transfused patients were 2.01; 95% CI: 1.56–2.59 and 1.59; 95% CI: 1.23–2.06, and 1.50; 95% CI: 1.15–1.97 and 1.24; 95% CI: 0.94–1.64 respectively. The division of transfused patients was done according to the storage time of the RBC unit with the highest storage time if the patient received more than one unit of RBC. The same pattern was found if transfused patients were divided according to the mean storage time of transfused blood.

**Fig 3:**
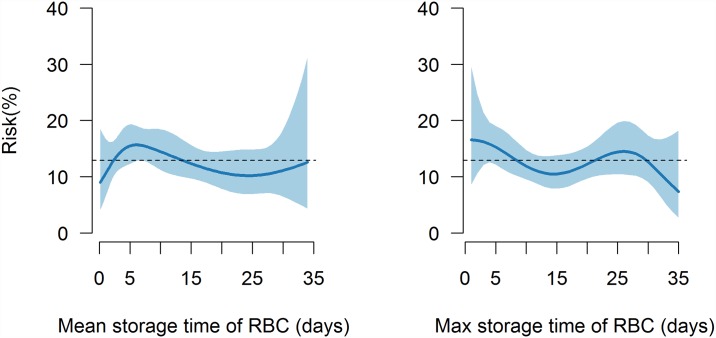
Risk for development of postoperative atrial fibrillation according to storage time of transfused red blood cell units. The baseline risk (dotted line) represents the risk when receiving 1–3 units of RBC, being male gender, being <60 years of age, receiving no medication (β-blocker, ACE-inhibitor, calcium antagonist), not having COPD or peripheral arteriosclerosis and when having a combined surgery as the surgery type.

Results of the analyses determining association between storage time of the RBC and the risk of developing POAF are shown in Fig 3, using maximal and mean storage times. Sensitivity analyses showed results robust towards change of placements of knots.

## Discussion

Allogeneic RBC transfusion was associated with an increased risk of POAF in a way that seems to be dose-dependent in this observational cohort study among patients undergoing cardiac surgery. This finding is in accordance with findings in other observational studies [24,25] although not supported by all studies [26]. However, increased storage time of the RBC prior to transfusion was not associated with an increased risk of POAF.

The majority of patients received less than five RBC units (Fig 1). However, some patients received >10 units on the day of surgery indicating severe bleeding problems. Especially, these patients may receive a mixture of RBC units with different storage time in the blood bank. The mean age of transfused blood was >10 days (Fig 2). Thus, patients will often receive a mixture of “fresh” and “old” blood if a cut-off value for “fresh” blood is defined as blood stored for <14 days. It seems as if prolonged storage of RBC units is associated with less risk of POAF compared with younger blood. Dividing transfused patients arbitrarily into two groups according to the storage time of a single transfused RBC unit (RBC stored for either <14 days or ≥14 days) or to either the mean and maximum storage age of transfused blood if patients received more than one unit of RBC. This finding is in accordance with previous surprising findings indicating that prolonged storage of blood may be beneficial compared with younger blood [19], although difficult to explain. In contrast to our findings, an observational study among 819 patients undergoing isolated CABG showed that POAF was significantly more often developed in transfused patients who received blood units all stored for >14 days compared with patients who received blood units all stored for <14 days [21]. Such different findings may be explained by e.g. differences in patient cohorts, different definitions of POAF and different methods for obtaining the diagnosis.

Several studies divided transfused blood into “fresh” or “old” blood” using a cut- off around 14 days [27]. This arbitrary cut-off is based on an increasing risk of storage lesions in stored RBC units over time [20]. These storage lesions may potentially reduce oxygen delivery to tissues and increase the inflammatory response following transfusion. We did not divide patients into groups who only received “young” or “old” blood in the present study as many patients will receive RBC units with a mixed storage time. Using storage time as a continuous variable, we were not able to identify any association between storage time of RBC and risk of POAF in patients receiving RBC.

As previous mentioned several predictors of POAF are well described [8–10] including allogeneic RBC transfusion [13,14]. In accordance with these findings, several of these risk factors were also more common in patients who developed POAF compared with the group of patients who did not develop POAF (Table 1). We did not find severe obesity (BMI >40) to be more prevalent among patients who developed POAF compared with non-obese patients. However, only few patients with severe obesity underwent surgery during the study period.

Although risk factors including RBC transfusion may predict development of POAF, these risk factors may not necessarily be the cause of POAF. Risk factors may just be markers for development of POAF, and therefore results must be evaluated with care. Theoretically, storage lesions in transfused blood may be a causal explanation for a potential increased risk of POAF due to the inflammatory response which transfused blood will create [15]. Thus, storage lesions may be an explanation for the positive association between RBC transfusion and development of POAF shown by other authors [13,14], but results from observational studies may be confounded by known and unknown risk factors that were not controlled for.

Only results from randomized studies should form the basis for any changes in clinical transfusion practice. Observational studies are only hypothesis generating. Until now, randomized studies and meta-analyses have not shown that transfusion of “young” RBC units are superior to transfusion with RBC with prolonged storage time regarding the risk of development of multiple organ dysfunction in patients undergoing cardiac surgery or among critically ill adult patients [18,19]. Thus, these studies do not support any changes in clinical practice with regard to the age of RBC units transfused, although POAF was not the outcome of interest in these studies.

The strengths of this study are the relative large cohort of consecutive patients from two university hospitals serving approximately one third of the population in Denmark, which is rather homogenous. Furthermore, it is a strength that we used a validated database containing prospectively recorded clinical data. Epidemiological studies are dependent on the validity regarding the data extracted from the databases used. The validity of a diagnosis of POAF in the WDHR from which we retrieved data regarding development of POAF was evaluated previously [28]. A positive predictive value (PPV) of 82.5% was calculated for a diagnosis of POAF in the WDHR. Reported PPVs of a diagnosis of atrial fibrillation are ranging from 70% to 96% when other administrative data sets are used [29], and although there is a risk of misclassification of patients with the regard to the outcome of interest, we find the validity of POAF data in the WDHR acceptable.

There are also limitation in relation to this study. In the present study, we excluded patients who received transfusions preoperative and/or later than the day of surgery in order to ensure that the outcome of interest developed after transfusion of RBC. However, there is still a risk of confounding in relation to the timing of transfusion and development of POAF, as we were not able to retrieve information on the date regarding development of POAF. POAF may have developed on the day of surgery and maybe before transfusion. However, we have no reason to believe that this would be the case in many patients as POAF most often develops between days 2–5 postoperative [30]. Another limitation is the methods used for obtaining a diagnosis of POAF. Continuous ECG monitoring which would have been the optimal method to obtain the diagnosis was not used in the patients until discharge, and the incidence of POAF in the present study may thus have been underestimated. Furthermore, POAF may develop after discharge and POAF in such cases are not recorded in the WDHR. However, we do not expect a potential misclassification to be related to the transfusion status of the patients. Prophylactic treatment with amidarone was administered to a minor subset of patients in Aarhus University Hospital. However, as the indication for this was not related to the risk of RBC transfusion, we do not believe that administration to some of the patients may have biased our results regarding development of POAF in relations to transfusion of RBC and storage time of the blood. Finally, we were not able to correlate our finding with the haemoglobin, haematocrit concentrations, pH and potassium concentration in the blood products transfused.

## Conclusion

In contrast to intraoperative allogeneic RBC transfusion in general, increased storage time of RBC is not associated with development of POAF in cardiac surgery.

## Supporting information

S1 File Supporting information file with all the underlying participant-level data (anonymous). (XLSX)
